# Exosomes derived from ccRCC cells confers fibroblasts activation to foster tumor progression through Warburg effect by downregulating PANK3

**DOI:** 10.1038/s41420-025-02434-8

**Published:** 2025-04-25

**Authors:** Yang Yang, Cheng Qiang, Zhu Jie, Han Ce, Huang Yan, Li Xiu-bin, Fan Wen-mei, Zhang Xu, Gao Yu

**Affiliations:** 1 Department of Urology, the third Medical Centre, Chinese PLA General Hospital, Beijing, China; 2 Medical School of Chinese PLA, Beijing, China

**Keywords:** Cancer metabolism, Renal cell carcinoma

## Abstract

The interaction between tumor-derived exosomes and stroma plays a crucial role in tumor progression. However, the mechanisms through which tumor cells influence stromal changes are not yet fully understood. In our study, through single-cell sequencing analysis of clear cell renal cell carcinoma tissues at varying stages of progression, we determined that the proportion of cancer-associated fibroblasts (CAFs) in advanced renal cell carcinoma tissues was notably higher compared to localized renal cell carcinoma tissues. Comparison of transcriptome sequencing and energy metabolism tests between CAFs primarily isolated from advanced renal cell carcinoma tissues and normal fibroblasts (NFs) revealed the occurrence of the Warburg effect during the fibroblast activation process. Additionally, we observed an increase in glucose transporter GLUT1 expression, total reactive oxygen species (ROS) levels, lactic acid production, and subsequent excretion of excess lactic acid through monocarboxylate transporter-4 (MCT4) in CAFs. Interestingly, renal cancer cells were found to uptake lactic acid via MCT1 upon interaction with CAFs, thereby enhancing their malignant phenotypes. Furthermore, the down-regulation of PANK3 induced by exosomes derived from renal cancer cells was identified as a crucial step in fibroblast activation. These findings indicate that exosomes play a role in facilitating intercellular communication between renal cancer cells and fibroblasts. Targeting this communication pathway could potentially offer new strategies for the prevention and treatment of advanced renal cell carcinoma.

## Introduction

Pulmonary metastasis and tumor thrombus are the most common aggressive progression of clear cell renal cell carcinoma (ccRCC) and the leading causes of cancer-related death [1, 2], and one of the reasons for this outcome is the intercellular communication between tumor and stromal cells in the microenvironment [3, 4]. In the fight against tumor progression, therapeutic strategies targeting microenvironment components have gained importance in recent years [5–9]. Among the various cell types in the tumor stroma, cancer-associated fibroblasts (CAFs) are the most abundant and play a crucial role in promoting tumor progression and metastasis [10]. CAFs are not a uniform cell type, two commonly used markers of CAFs are α-smooth muscle actin (α-SMA) and fibroblast activation protein (FAP) [11, 12]. The interaction between tumor cells and CAFs has been extensively researched. However, the specific mechanism through which tumor cells activate fibroblasts is not yet clearly understood, even less in ccRCC progression.

First discovered in the 1980s, exosomes are believed to be bilayer vesicles with a diameter ranging from 50 to 150 nm [13, 14]. These tiny structures can be produced by various cell types and are released into the extracellular environment through fusion with the cell membrane. Exosomes are identified by the presence of highly enriched specific proteins like TSG101, CD63, HSP70, CD9, and CD81 [15–17]. These vesicles have been found to play a crucial role in transmitting genetic materials, activating signaling pathways, inducing metabolic reprogramming, influencing tumor progression, and modulating tumor response to drugs and immune cells [18–24]. However, the relationship between tumorigenic exosomes and the progression of ccRCC remains unclear. In addition, as one of the most extensively studied malignant tumors, ccRCC involves mutations in a variety of genes that impact cell metabolism and is recognized as a metabolism-related disease. This study aims to investigate the causes of ccRCC progression through intercellular communication and metabolic reprogramming. Transcriptome sequencing tests between CAFs and NFs revealed the occurrence of the Warburg effect and the target gene PANK3. Furthermore, the activated fibroblasts were found to promote tumor progression by increasing the secretion of lactic acid and inflammatory cytokines. The crosstalk between stromal and tumor cells revealed a new mechanism of ccRCC progression and provides a potential therapeutic strategy for advanced ccRCC, including lung metastasis and tumor thrombus.

## Results

### Advanced ccRCC tissues are enriched with CAFs

**Fig. 1:**
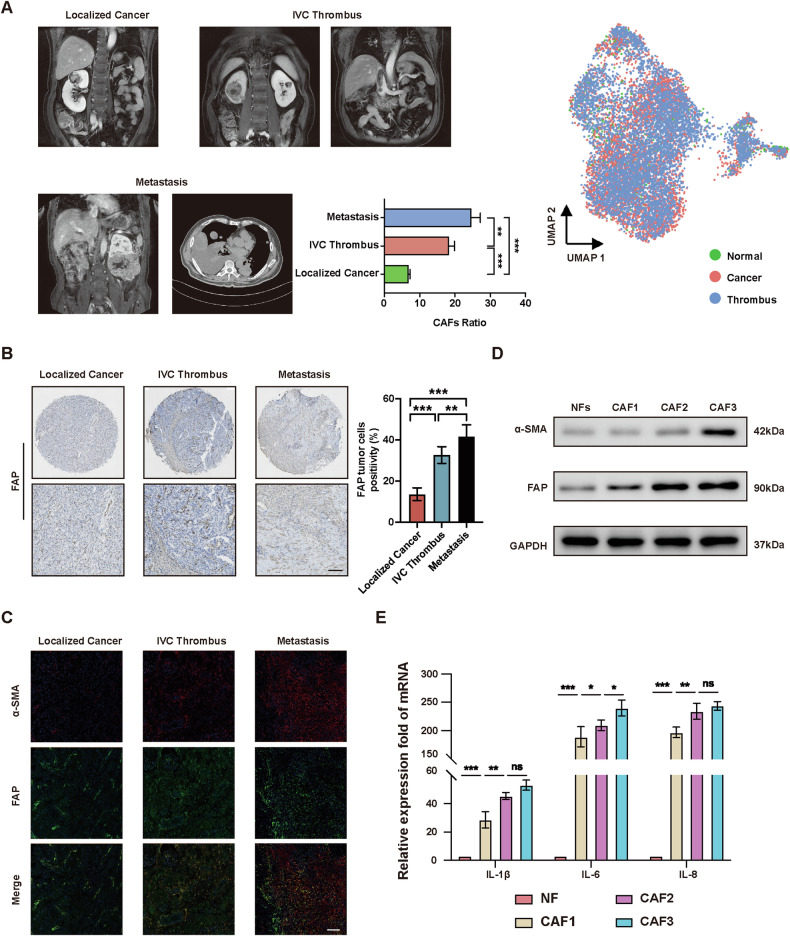
Advanced ccRCC tissues are enriched with CAFs. **A** UMAP plot of CAFs derived from ccRCC tissues at different stages of progression, colored by tissue origin. **B** IHC staining detection of FAP on ccRCC tissues. Scale bar, 50 μm. **C** Immunofluorescence detection of α-SMA and FAP on ccRCC tissues. Scale bar, 100 μm. **D** Western blotting assay of α-SMA and FAP in primary isolated NFs and CAFs. **E** qRT-PCR analysis of pro-inflammatory genes expression of NFs and CAFs. Each experiment was performed three times independently and results are presented as mean ± s.d. Student’s *t*-test was used to analyze the data (*p < 0.05; **p < 0.01; ***p < 0.001).

To investigate the relationship between cancer-associated fibroblasts (CAFs) and the progression of renal cell carcinoma, we obtained primary tumor tissue samples from three distinct patient groups: those with localized renal cell carcinoma, renal cell carcinoma with inferior vena cava (IVC) tumor thrombus, and renal cell carcinoma with lung metastasis. Through single-cell RNA sequencing (scRNA-seq), we observed notable differences in cell composition among the three groups, particularly a significantly higher proportion of CAFs in advanced renal cell carcinoma compared to localized cases (Fig. 1A). Immunohistochemical staining and Immunofluorescence of CAFs markers were also performed on the primary tumor tissue samples, which further confirmed that the ratio of CAFs increased in ccRCC tissues with progression (Fig. 1B, C). To comprehend the distinctions among CAFs obtained from primary ccRCC tissues at various stages of progression, we defined CAF1 as CAFs derived from localized ccRCC tissues, CAF2 from ccRCC tissues with IVC tumor thrombus, and CAF3 from ccRCC tissues with lung metastasis. Compared with normal fibroblasts (NFs), we found that as the progression of ccRCC advanced, the expression levels of α-SMA and FAP proteins in primary isolated CAFs also increased (Fig. 1D). The aforementioned findings demonstrate that CAFs can express pro-inflammatory genes at high levels, leading to the formation of an inflammatory microenvironment that promotes tumor progression (Fig. 1E). These findings collectively reveal that CAFs are associated with progression of ccRCC.

### Tumor-promoting capability of CAFs demonstrates variability

**Fig. 2:**
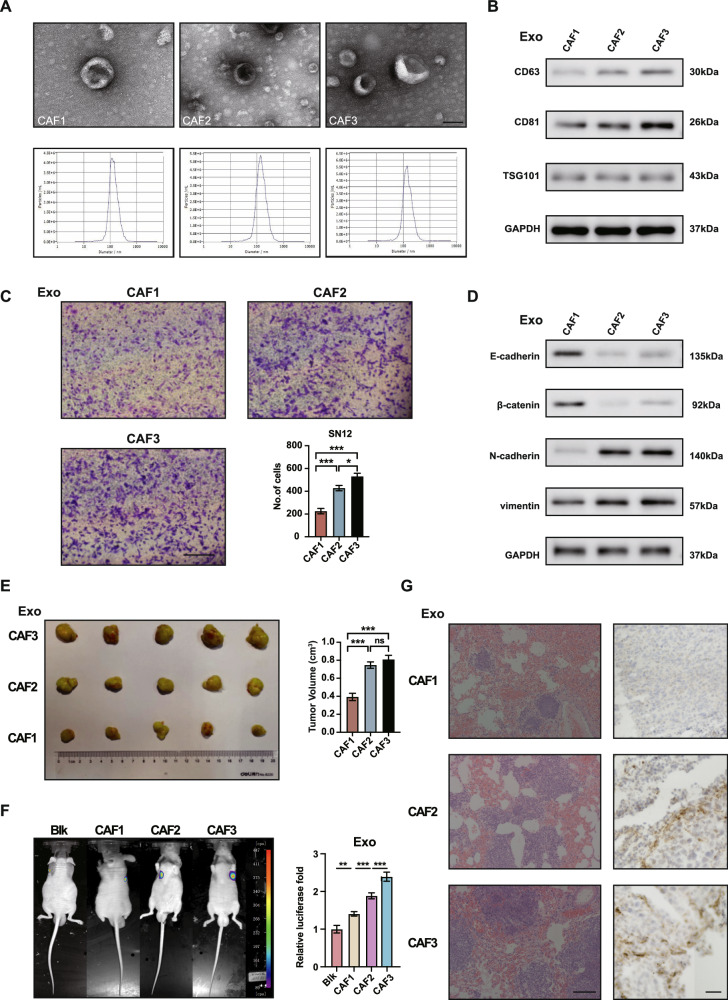
Tumor-promoting capability of CAFs demonstrates variability. **A** Electron microscopy and Nanosight particle tracking analysis of exosomes derived from CAFs. Scale bar, 100 nm. **B** Western blotting assay of indicated proteins in exosomes from CAFs. **C** Migration assays of SN12 treated with equal quantities of exosomes derived from CAFs. Scale bar, 100 μm. **D** Immunoblotting assays of EMT markers in SN12 treated with equal quantities of exosomes derived from CAFs. **E** Xenograft assays of SN12 with indicated treatments were performed on nude mice. Representative tumors (left) and tumor volumes (right) were shown. **F** Representative images and quantitative analysis of lung metastasis of indicated mice treated with exosomes derived from CAFs or blank control were determined by luciferase-based bioluminescence imaging. **G** HE and IHC staining of FAP on lung metastatic tissue samples of indicated nude mice. Scale bar, 50 μm. Each experiment was performed three times independently and results are presented as mean ± s.d. Student’s *t*-test was used to analyze the data (*p < 0.05; **p < 0.01; ***p < 0.001).

Numerous studies have shown that α-SMA (+) FAP (+) CAFs play a role in promoting tumor progression [25, 26]. To delve deeper into the variations in intracellular communication between tumor cells and CAFs, we conducted experiments using primary isolated CAFs from ccRCC tissues at different stages of progression. Exosomes were extracted from the conditioned medium using ultracentrifugation and were observed to have a ‘teacup shape’ under a transmission electron microscope. We utilized Nanosight particle tracking analysis to determine that the exosomes from the indicated conditioned medium were mostly within the 100–150 nm range. The concentration of exosomes derived from CAF2 and CAF3 was found to be enriched at a higher level than those from CAF1(Fig. 2A). Additionally, the presence of characteristic exosomal markers CD63, CD81, and TSG101 provided further confirmation that the isolated particles were indeed exosomes (Fig. 2B). Transwell assays indicated that compared with CAF1, exosomes derived from CAF2 and CAF3 significantly improved the migration ability of the SN12 cells (Fig. 2C).

Notably, CAF2-and CAF3-derived exosomes exhibit a greater capacity to promote EMT in renal cancer cells and tumorigenesis in vivo (Fig. 2D, E). Additionally, the exosomes from CAF2 and CAF3 were also found to enhance the development of lung metastases caused by SN12 cells (Fig. 2F). FAP staining via immunohistochemistry in lung metastases indicated higher levels of cancer-associated fibroblasts (CAFs) in the CAF2 and CAF3 groups (Fig. 2G). These findings provide further evidence that CAFs derived from advanced ccRCC exhibit enhanced tumor-promoting capabilities.

### CAFs undergo the Warburg effect in response to activation

**Fig. 3:**
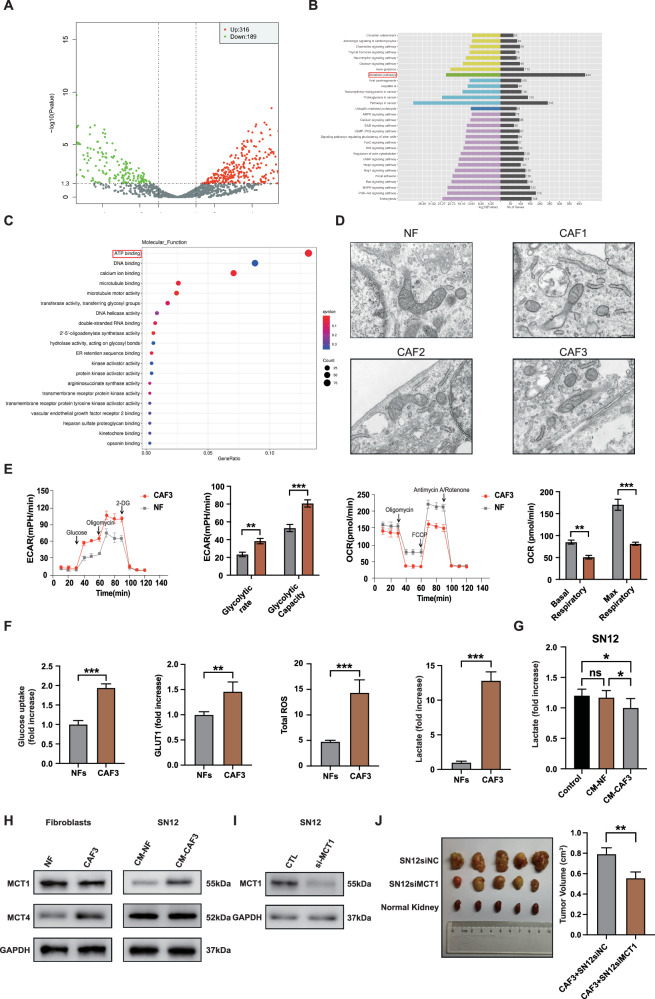
CAFs undergo the Warburg effect in response to activation. **A** Transcriptome sequencing of differentially expressed genes between CAFs and NFs. **B** GO pathway enrichment analysis of differentially expressed genes. **C** KEGG pathway enrichment analysis of differentially expressed genes. **D** Transmission electron microscopy images of NFs and CAFs. Scale bar, 50 μm. **E** Analysis of ECAR and OCR between CAF3 and NF. **F** Comparison of glucose uptake, GLUT1 expression, mitochondrial ROS level, and lactate production between NF and CAF3. **G** Detection of lactic acid in indicated conditioned medium. **H** Immunoblotting assays of MCTs in NFs and SN12 with indicated treatments. **I** Immunoblotting assays of MCT1 expression in NFs transfected with siRNAs targeting MCT1 or control. **J** Xenograft assays of SN12 with indicated treatments were performed on nude mice. Representative tumors (left) and tumor volumes (right) were shown. Each experiment was performed three times independently and results are presented as mean ± s.d. Student’s *t*-test was used to analyze the data (*p < 0.05; **p < 0.01; ***p < 0.001).

To delve deeper into the distinctions between CAFs and NFs, we analyzed three sets of primary CAFs and NFs and compared their gene expression patterns. Our analysis revealed that 316 genes were up-regulated while 189 genes were down-regulated in CAFs. Subsequent GO analysis and KEGG pathway enrichment analysis on these genes with notable alterations indicated that the differentially expressed genes primarily impacted metabolic pathways and ATP binding processes (Fig. 3A–C). Additionally, we utilized transmission electron microscopy to observe NFs and CAFs. The overall and mitochondrial structure of CAFs were found to be similar. Compared to NFs, the CAFs exhibited slight edema, the cell matrix was sparse, the mitochondria were swollen, and the cristae structure was less pronounced (Fig. 3D). Subsequently, the oxygen consumption rate (OCR) and extracellular acidification rate (ECAR) of high tumor-promoting CAF3 and NFs were compared using the Seahorse Bioscience XFe96 Extracellular Flux Analyzer platform. The results indicated that CAF3 exhibits a higher glycolytic rate and capacity and lower basal and max respiratory levels, indicating a pronounced reliance on the Warburg effect (Fig. 3E). In addition, CAF3 exhibited heightened glucose uptake capacity through upregulation of GLUT1 glucose transporter. Furthermore, CAF3 demonstrated elevated basal ROS levels and an over tenfold increase in lactic acid production (Fig. 3F). Then, SN12 cells were cultured with DMEM serum-free medium, NFs conditioned medium, and CAF3 conditioned medium, respectively. The level of lactic acid in the conditioned medium of CAF3 decreased significantly, suggesting that SN12 cells may consume lactic acid (Fig. 3G). Monocarboxylate transporters (MCTs) are key molecules in glycolysis, responsible for the transport of lactic acid. MCT4 is mainly responsible for the excretion of intracellular lactic acid, while MCT1 is responsible for the uptake of lactic acid into the cell [27]. CAF3 expressed higher levels of MCT4, and SN12 cells co-cultured with conditioned medium of CAF3 expressed higher levels of MCT1. The findings indicate that the activation of fibroblasts leads to a significant production of lactic acid. Additionally, co-cultured SN12 cells are capable of taking up lactic acid into the cell through MCT1 (Fig. 3H). In vivo experiments further demonstrated that silencing MCT1 resulted in the inhibition of tumor growth in an orthotopic transplantation tumor model, highlighting the impact of lactic acid uptake on the tumorigenicity of renal cancer cells (Fig. 3I, J).

### The down-regulation of PANK3 induces fibroblast activation

**Fig. 4:**
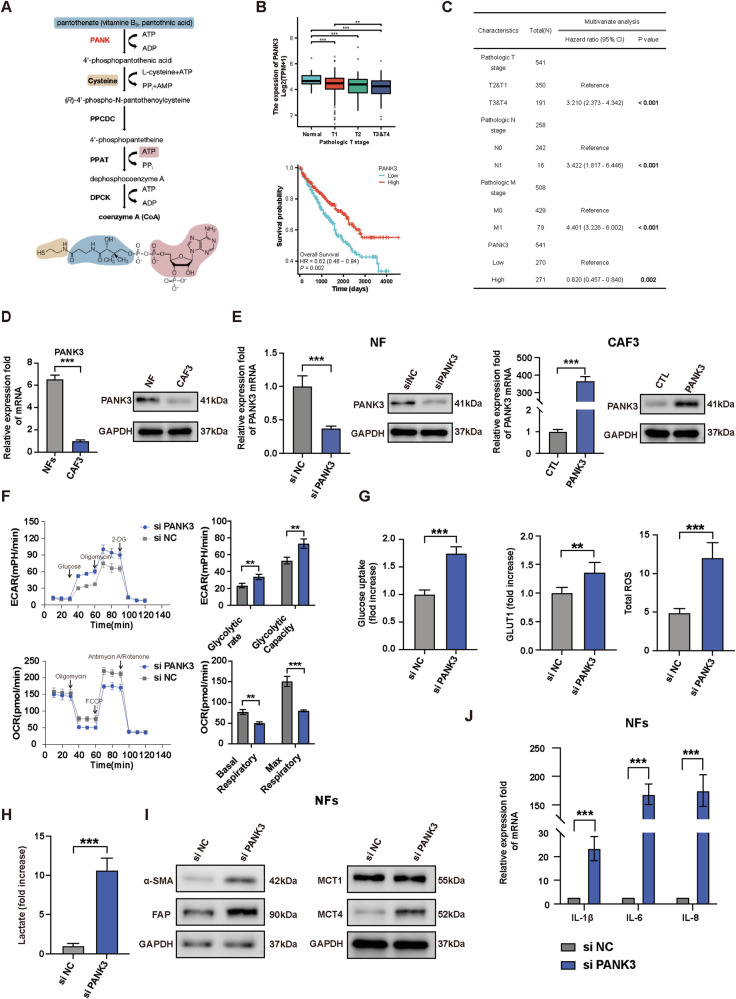
The down-regulation of PANK3 induces fibroblast activation. **A** The process of synthesizing Coenzyme A and key enzymes. **B** Differential expression and prognostic analysis of PANK3 in the TCGA-KIRC database. **C** Multivariate COX regression analysis of PANK3 in the TCGA-KIRC database. **D** qRT-PCR and immunoblotting assays of PNAK3 in NF and CAF3. **E** qRT-PCR and immunoblotting assays of PANK3 in NF and CAF3 transfected with si PANK3 or plasmid vector containing PANK3. **F** Analysis of ECAR and OCR of NFs transfected with si PANK3 or si NC. **G** Comparison of glucose uptake, GLUT1 expression, total ROS level of NFs transfected with si PANK3 or si NC. **H** Comparison of lactate production of NFs transfected with si PANK3 or si NC. **I** Immunoblotting assays of α-SMA, FAP, and MCTs in NFs transfected with si PANK3 or si NC. **J** qRT-PCR analysis of pro-inflammatory genes expression of NFs transfected with si PANK3 or si NC. Each experiment was performed three times independently and results are presented as mean ± s.d. Student’s t-test was used to analyze the data (*p < 0.05; **p < 0.01; ***p < 0.001).

In the transcriptome sequencing results of the CAFs compared with NFs, the top 10 genes with different fold changes were BBC3, DCAF3, GNAI3, HIPK2, PANK3, PHACTR4, PPP2R2A, STOX2, TLE3, and ZNF652. Notably, PANK3 showed enrichment in multiple key pathways, as well as being significantly enriched in both GO analysis and KEGG pathway enrichment analysis. Pantothenate kinase 3 (PANK3) plays a crucial role as a key enzyme in the de novo synthesis of coenzyme A (Fig. 4A), participating in a diverse array of cellular metabolic processes. Analysis of tissue samples from TCGA-KIRC patients revealed that the expression of PANK3 in ccRCC tissues was significantly lower than that in adjacent normal tissues. Furthermore, PANK3 expression decreased with the advancement of the pathological T stage. Additionally, Kaplan–Meier survival curves indicated that higher PANK3 expression was correlated with improved survival outcomes (Fig. 4B). Subsequent Cox multivariate analysis adjusted for pathological T stage and M stage, validating that the expression level of PANK3 serves as an independent prognostic predictor (HR: 0.620, 95% CI: 0.457–0.840; *p* = 0.002) (Fig. 4C). Consistent with our hypothesis, the expression of PANK3 in CAF3 cells was notably lower in comparison to NFs (Fig. 4D). si PANK3 and PANK3 overexpression plasmids were separately transfected into NFs and CAF3, and the levels of PANK3 were verified through qRT-PCR and western blot analysis (Fig. 4E). NFs transfected with si PANK3 exhibits a higher glycolytic rate and capacity and lower basal and max respiratory levels, increased glucose uptake and total ROS, as well as a significant increase in lactic acid production (Fig. 4F–H). Additionally, NFs transfected with si PNAK3 exhibited elevated expression levels of CAF surface markers (α-SMA, FAP) and MCT4, along with heightened inflammatory factors to construct an inflammatory microenvironment (Fig. 4I, J). These findings collectively reveal that the down-regulation of PANK3 mediates fibroblast activation.

### Tumor-derived exosomal miR-222-3p directly targets PANK3 in fibroblasts

**Fig. 5:**
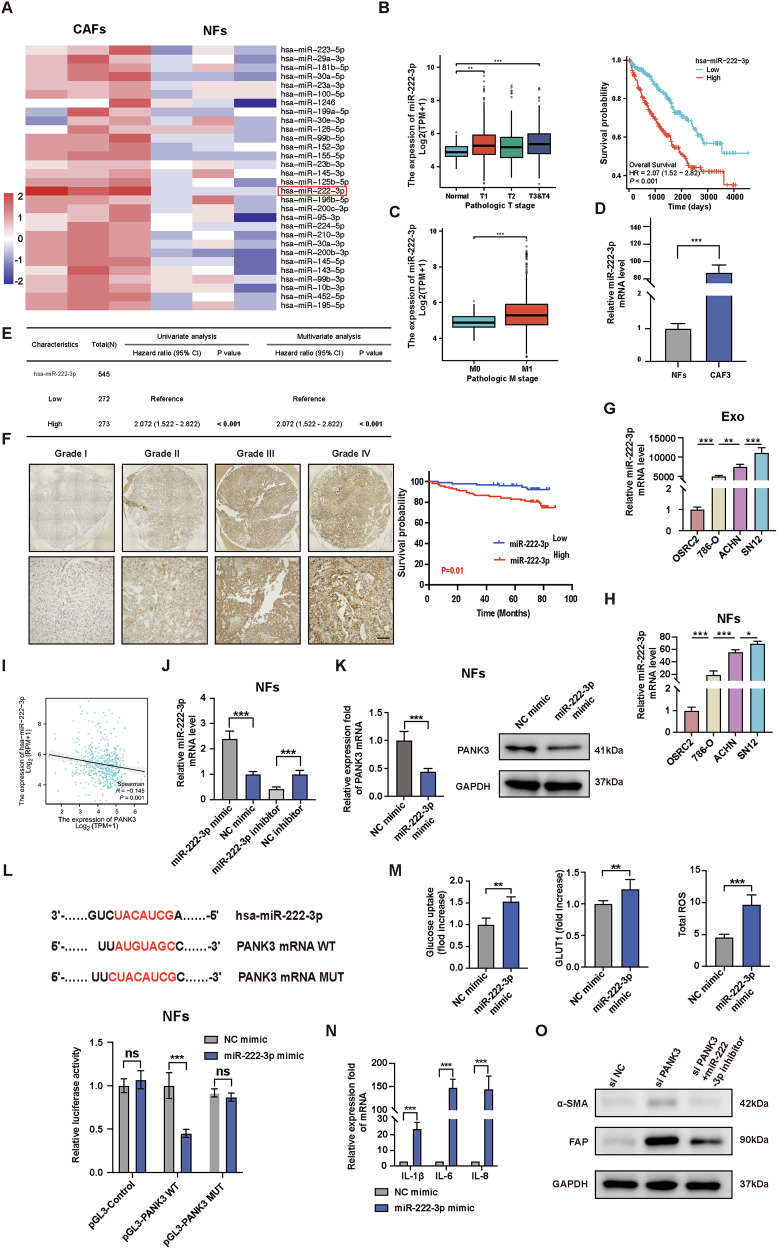
Tumor-derived exosomal miR-222-3p directly targets PANK3 in fibroblasts. **A** MiRNAs sequencing of differentially expressed genes between CAFs and NFs. **B** Differential expression and prognostic analysis of miR-222-3p in the TCGA-KIRC database. **C** Differential expression of miR-222-3p in patients with or without metastasis in the TCGA-KIRC database. **D** qRT-PCR assays of miR-222-3p in NF and CAF3. **E** Univariate and multivariate COX regression analysis of miR-222-3p in the TCGA-KIRC database. **F** In situ hybridization of miR-222-3p on human ccRCC tissues. Scale bar, 50 μm. **G** Exosomal miR-222-3p expression from different ccRCC cell lines. **H** qRT-PCR analysis of miR-222-3p expression of NFs treated with equal quantities of exosomes derived from cRCC cell lines. **I** Correlation analysis of miR-222-3p and PANK3 expression in the TCGA-KIRC database. **J** Alteration of miR-222-3p levels in NFs following transfection with miR-222-3p mimic or miR-222-3p inhibitor. **K** qRT-PCR and immunoblotting assays of PANK3 in NFs transfected with miR-222-3p mimic or NC mimic. **L** Relative luciferase activity of NFs in the presence of indicated treatments. **M** Comparison of glucose uptake, GLUT1 expression, total ROS level of NFs transfected with miR-222-3p mimic or NC mimic. **N** qRT-PCR analysis of pro-inflammatory genes expression of NFs transfected with miR-222-3p mimic or NC mimic. **O I**mmunoblotting assays of α-SMA and FAP in NFs with indicated treatments. Each experiment was performed three times independently and results are presented as mean ± s.d. Student’s *t*-test was used to analyze the data (*p < 0.05; **p < 0.01; ***p < 0.001).

**Fig. 6:**
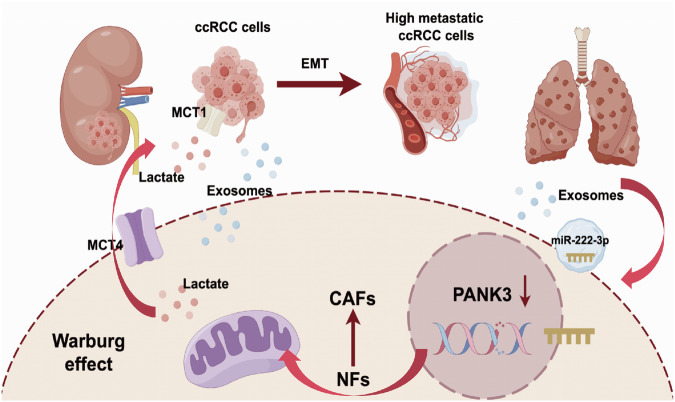
Proposed schematic diagram of tumor-derived exosomes promoting the progression of renal cell carcinoma by down-regulating PANK3 to activate fibroblasts.

Numerous studies have demonstrated that tumor-derived exosomal miRNAs trigger fibroblast activation [28–30]. To investigate the potential upstream regulators of PANK3, we conducted miRNA sequencing on three pairs of primarily isolated CAFs and NFs, identifying the top 30 miRNAs. Interestingly, our findings revealed that out of these 30 miRNAs, only miR-222-3p exhibited all high expression levels in CAFs and all low expression levels in NFs (Fig. 5A). The analysis of tissue samples from TCGA-KIRC patients revealed that miR-222-3p expression in ccRCC tissues was higher compared to adjacent normal tissues. Furthermore, as the pathological T stage increases, the difference in miR-222-3p levels between tumor tissues becomes more pronounced compared to adjacent normal tissues. Additionally, the Kaplan–Meier survival curve indicated that a lower expression of miR-222-3p was associated with improved survival outcomes (Fig. 5B). In addition, the expression level of miR-222-3p in tissues of patients with metastatic ccRCC was higher, which aligned with the findings from our primary isolated CAF3 cell line (Fig. 5C, D). To further explore the correlation between miR-222-3p levels and clinicopathological features of ccRCC, we conducted miR-222-3p in situ hybridization on 206 localized or progressive ccRCC tissues. The specimens were then categorized into two groups based on their miR-222-3p expression score, namely low and high miR-222-3p expression groups. According to follow-up results, high miR-222-3p expression predicted poor survival outcomes, which was consistent with the multivariate COX regression result of the TCGA-KIRC database (Fig. 5E, F). To investigate if the levels of exosomal miR-222-3p in ccRCC cell lines are associated with varying metastatic abilities, we selected four renal cancer cell lines, including high-metastatic cell lines SN12 and ACHN, and low-metastatic cell lines OSRC2 and 786-O, and qRT-PCR analysis was conducted to validate the expression level of miR-222-3p in exosomes derived from various metastatic background renal cancer cell lines. The findings revealed that the level of miR-222-3p in exosomes from cell lines with metastatic background was significantly higher compared to those without (Fig. 5G). The level of miR-222-3p in normal fibroblasts (NFs) cultured with exosomes from the mentioned four cell lines exhibited a consistent pattern (Fig. 5H). The tissue data from TCGA-KIRC patients revealed a negative correlation between the levels of miR-222-3p and PNAK3 (Fig. 5I). In order to investigate the presence of a direct targeted regulation between miR-222-3p and PANK3, we transfected miR-222-3p mimic and inhibitor into NFs and verified the levels of miR-222-3p through qRT-PCR (Fig. 5J). Subsequently, we confirmed that miR-222-3p reduces the expression of PANK3 expression in NFs (Fig. 5K). Then we compared the PANK3 full-length sequence with the miR-222-3p sequence and found that the PANK3 coding sequence could be the target of miR-222-3p. To confirm this, the luciferase vectors containing the binding sites for wild-type and mutant miR-222-3p were cloned and analyzed. The results revealed a significant decrease in luciferase activity in cells transfected with the wild-type binding site vector NFs when miR-222-3p was present. In contrast, cells transfected with the mutant binding site vector did not exhibit this repression (Fig. 5L). NFs transfected with miR-222-3p also exhibit increased glucose uptake, total ROS, and heightened inflammatory factors (Fig. 5M, N). Moreover, The upregulation of CAF markers caused by si PANK3 transfection can be counteracted by the transfection of a miR-222-3p inhibitor (Fig. 5O). Overall, these findings suggest that tumor-derived exosomal miR-222-3p directly targets PANK3 and regulates fibroblast activation. The proposed schematic diagram is presented in Fig. 6.

## Discussion

The tumor microenvironment is a complex system that relies on intercellular communication, and changes in this system are closely linked to tumor progression and metastasis [31, 32]. Recent studies have shown that exosomes play a crucial role in intercellular communication [19, 20]. Therefore, it is essential to investigate the interaction between exosome-mediated tumor cells and mesenchymal cells. In our study, we examined exosomes derived from CAFs obtained from primary ccRCC tissues at varying stages of progression. Our findings indicate that CAFs isolated from primary ccRCC tissues of patients with advanced stages, including those with IVC tumor thrombus or lung metastasis, secrete a higher quantity of exosomes that exhibit enhanced migratory capabilities, tumorigenicity, and the ability to promote distant metastasis in renal cancer cells. Additionally, these exosomes are found to induce EMT in renal cancer cells. Furthermore, exosomal miR-222-3p derived from renal cancer cells is shown to induce the transformation of fibroblasts into CAFs by down-regulating the target gene PANK3 and inducing metabolic reprogramming. CAFs, in turn, drive the progression of renal cell carcinoma through increased production of lactic acid via aerobic glycolysis. The elevated levels of miR-222-3p in renal cancer tissues have also been shown to be associated with a negative prognosis for patients, highlighting the importance of developing targeted prevention and treatment strategies.

PANK is recognized as the primary enzyme that limits the rate of the coenzyme A biosynthetic pathway [33]. Previous research has identified that a high expression of the PANK3 gene serves as a biomarker for a favorable prognosis in hematopoietic system tumors [34]. However, the involvement of PANK3 in solid tumors remains relatively understudied. To investigate the impact of PANK3 on fibroblast activation, we utilized si PANK3 to reduce PANK3 levels in NFs. Our findings revealed alterations in the metabolic profile of fibroblasts with decreased PANK3, characterized by reduced basic and maximum OCR alongside enhanced glycolysis. Furthermore, the expression of CAF surface markers was elevated in PANK3-deficient NFs, accompanied by increased lactic acid production, collectively suggesting a crucial role for PANK3 in driving fibroblast activation.

Glucose is a vital nutrient for human cell survival, as various cellular reactions depend on carbohydrate energy supply. Typically, cells generate a significant amount of ATP through the mitochondrial respiratory chain pathway. However, malignant tumors exhibit a distinct metabolic behavior compared to normal tissues. Even in oxygen-rich environments, tumor cells tend to preferentially produce energy by converting pyruvate to lactic acid [35]. Additionally, these cells require other biological macromolecules like nucleotides and proteins for processes such as division and proliferation, which can be sustained under this altered metabolic model [36]. This metabolic shift, known as the Warburg effect, is commonly observed in malignant tumors across different tissues. Our study revealed that during the transition of fibroblasts to CAFs, aerobic glycolysis takes place, leading to the production of a significant amount of lactic acid. Activated fibroblasts release lactic acid through MCT4, which can then be taken up by co-cultured renal cancer cells via MCT1, establishing a ‘lactic acid shuttle’ between CAFs and renal cancer cells. Furthermore, inhibiting the expression of MCT1 in renal cancer cells has been shown to impact tumor growth in the orthotopic transplantation tumor model of renal cancer cells in nude mice. This metabolic interaction between tumor cells and CAFs highlights how they mutually promote metabolic reprogramming and drive the progression of renal cancer.

In conclusion, our findings demonstrate that tumor-derived exosomal miR-222-3p induces aerobic glycolysis and prompts fibroblast activation into CAFs by downregulating PANK3. Furthermore, CAFs contribute to increased tumorigenicity and lung metastasis of tumor cells by releasing exosomes, lactic acid, and inflammatory cytokines. Notably, the elevated miR-222-3p expression in ccRCC tissues correlates with a poorer patient prognosis. This study unveils a novel molecular mechanism underlying the interaction between tumor cells and fibroblasts in promoting tumor progression, offering potential insights for the prevention and treatment of advanced renal cell carcinoma.

## Materials and methods

### Single-cell suspension preparation and sequencing

Single-cell suspensions for single-cell RNA-seq were prepared through mechanical and enzymatic dissociation. The Single Cell 3′ Library and Gel Bead Kit V3.1 and Chromium Single Cell B Chip Kit were utilized following the manufacturer’s instructions to generate barcoded scRNA-seq. Subsequently, the libraries underwent sequencing on an Illumina NovaSeq6000 sequencer, targeting a sequencing depth of at least 100,000 reads per cell with a paired-end 150 bp (PE150) reading strategy (conducted by CapitalBio Technology, Beijing).

### Specimens

A tissue microarray was obtained from the Chinese PLA General Hospital, consisting of localized or progressive ccRCC tissue samples from 206 patients. The clinicopathological features of the patients are presented in Supplementary Table 1. All procedures were approved by the Ethics Committee of Chinese PLA General Hospital and informed consent was obtained before the study.

### Primary CAFs

The renal cancer tissue was placed in PBS, minced with sterile scissors, and transferred to a 50 ml centrifuge tube. Concurrently, 10 ml of collagenase was added, and the mixture was digested at 37 °C for 1 h. The resulting cell mixture was filtered through a 70 μm pore size filter and placed in a new centrifuge tube, where it was centrifuged at 200×*g* for 3 min. The supernatant was discarded, and 10 ml of PBS was added to the pellet, which was then thoroughly mixed and centrifuged again at 200×*g* for 3 min. After discarding the supernatant, the cells were resuspended in DMEM/F12 supplemented with 10% FBS (Pricella) and cultured in 6 cm dishes. The adherence of the cells was observed daily, noting that CAFs exhibited a strong ability to adhere to the dish surface. Throughout the culture period, the dishes were repeatedly washed with sterile PBS to remove non-adherent cells, and the cells were cultured for a total of 2 weeks, during which their status was closely monitored. The isolated CAFs were identified by the detection of specific markers, including α-SMA and FAP, using immunofluorescence.

### Cell culture

Human renal carcinoma cell lines OSRC2, 786-O, ACHN, and SN12 were obtained from the Cell Bank of Type Culture Collection of the Chinese Academy of Sciences. The cells were cultured in 1640, 1640, MEM, and DMEM (Pricella), respectively, with the addition of 10% FBS (Pricella). The primary normal fibroblasts (NFs) were obtained from normal kidney tissue and cultured in a specialized medium (Pricella, CM-H072) at 37 °C in a humidified incubator with 5% CO_2_. The cell lines were identified using short tandem repeats (STR) analysis and confirmed to be free of mycoplasma contamination.

### Reagents and antibodies

Antibodies for TSG101 (ab125011, 1:1000), CD81 (ab79559, 1:1000), and α-SMA (ab7817, 1: 2000) were purchased from Abcam. Antibody for CD63 (A5271, 1:1000) was purchased from ABclonal. Antibodies for FAP (66562s, 1:1000), and GAPDH (5174S, 1:1000) were purchased from Cell Signaling Technology. Antibody for PANK3 (bs-8339R, 1:1000) was purchased from Bioss. Antibodies for MCT1 (abs120479, 1:1500) and MCT4 (abs124388, 1:2000) were purchased from Absin.

### Western blotting

Whole-cell protein extracts were homogenized in cell lysates and centrifuged at 12,000 rpm for 15 min to obtain the protein concentration, which was measured using the bicinchoninic acid (BCA) assay. The protein was then transferred onto a nitrocellulose filter membrane and incubated with a specific antibody. The immune complex was subsequently incubated with the corresponding secondary antibody and detected using the Tanon chemiluminescence imaging system.

### RNA extraction and qRT-PCR

Total RNA was extracted using an RNA-Quick Purification Kit (ES science, RN001) according to the manufacturer’s instructions. Reverse transcription was performed using Fast All-in-One RT Kit (ES science, RT001). Real-time PCR was conducted using Super SYBR Green qPCR Master Mix (ES science, QP002). For miRNAs, reverse transcription was performed using a microRNA Reverse Transcription Kit (ES science, EZB-miRT4-L). Real-time PCR was conducted using EZ-Probe qPCR Master Mix for microRNA (ES science, EZB-miprobe-R2). The sequences of all indicated primers were listed in Supplementary Table 2.

### RNA interference and plasmids

siRNAs (si NC, siRNA targeting miR-222-3p or PANK3) and mimics of the miRNAs mentioned were obtained from Ribobio Company (Guangzhou, China). The sequences of the siRNAs and miRNA mimic are listed in Supplementary Tables 2, 3. The plasmid vector containing PANK3 and an empty vector were provided by Omiget Technology (Beijing, China).

### Animal studies

In order to study the impact of exosomes on lung metastasis, 15 male nude mice (4-week-old) were purchased from Beijing Vital River Laboratory Animal Technology. We injected 1 × 10^6^ luciferase-labeled SN12 cells into mice via tail vein. Subsequently, the mice were randomly and blindly assigned to groups, and the exosomes from various fibroblast-conditioned media were standardized to a concentration of 100 μg per 100 μl. Based on the group assignments, 100 μl of exosomes from the designated CAFs-conditioned medium were administered via tail vein injection twice a week for a duration of 6 weeks. The Tanon imaging system was utilized to analyze the resulting lung metastasis.

For xenograft assays, 10 male nude mice (4-week-old) purchased from Beijing Vital River Laboratory Animal Technology were randomly and blindly divided into SN12si NC, and SN12siMCT1 (*n* = 5 per group). 1 × 10^6^ educated SN12 cells were injected into the renal capsule of nude mice. The size of the tumors was measured at specific times. All animal experiments were approved by the Committee on Use and Care of Animals of Chinese PLA General Hospital.

### Migration assay

Matrigel diluent was used to coat the upper surface of the membrane at the bottom of the Transwell chamber, and the cells prepared were cultured in serum-free medium for 12 h in advance. The cell density was adjusted to 1 × 10^5^/ml. Add 500 μl complete medium to the lower chamber of Transwell, and add 200 μl cell suspension prepared in front of the upper chamber. The cell migration ability was cultured in a CO_2_ incubator. Staining was performed using a crystal violet solution, and the number of cells was averaged by randomly selecting three fields.

### Glucose uptake

Cells were treated with CM for 72 h. 2-deoxy-glucose uptake was evaluated in a buffered solution containing 0.5 mCi/mL [3H]deoxyglucose for 15 min at 37 °C. Subsequently, cells were washed with cold PBS, lysed with 0.1 mol/L NaOH, and the incorporated radioactive material was assayed by liquid scintillation counting.

### ROS evaluation

Cells were treated with 5 mmol/L DCF-DA for 3 min and then lysed using RIPA buffer. The fluorescence values were normalized to protein content. Evaluation of mitochondrial ROS was conducted by adding 5 mmol/L Mitosox to the cells for 15 min at 37 °C. Following a wash with PBS, fluorescence was analyzed using a cytofluorimeter.

### Immunohistochemistry and in situ hybridization analysis

For immunohistochemistry, after incubating with primary antibodies, the slides were subjected to procedures utilizing the Immunohistochemistry Kit (Biosharp) in accordance with the manufacturer’s instructions. For in situ hybridization analysis, the slides underwent treatment using the hsa-miR-222-3p miRCURY LNA detection probe and the Enhanced Sensitive IDH Detection Kit (Boster). The resulting images were captured and assessed using TissueFAXS software.

### Immunofluorescence analysis

CAFs and NFs were plated on the coverslip and cultured overnight under 37 °C, and then fixed in 4% paraformaldehyde for 30 min. Cells were incubated with 5% BSA for 1 h after three times of washes. Primary antibodies targeting α-SMA (1:2000, Abcam, #ab7817, USA) and FAP (1:1000, Cell Signaling Technology, #66562s, USA) were used. After incubation with indicated primary antibodies overnight, Alexa Fluor 488 or 546-labeled secondary antibodies (1:200, Abcam) were used to probe primary antibodies for 2 h.

### Isolation and analysis of exosomes

Exosome-free serum was incorporated into the cell culture medium configuration. Exosomes were extracted from the conditioned medium through traditional ultra-speed differential centrifugation. The exosomes were then observed through transmission electron microscopy and quantified using Nano-Sight NS300.

### Luciferase reporter assay

Ribobio Company designed and synthesized the dual luciferase reporter vector with the renilla luciferase gene (hRluc) as the reporter fluorescence and the firefly luciferase gene (hluc) as the corrected fluorescence. The 3’UTR region of the gene was cloned downstream of the hRluc gene, and the miRNA was co-transfected with the reporter gene vector. The down-regulation of the relative fluorescence value of the reporter gene confirmed the interaction between the miRNA and the target gene.

### Measurement of lactate production, OCR and ECAR

Lactate Colorimetric Assay Kit (Sigma-Aldrich) was used according to the manufacturer’s instructions. The Seahorse Bioscience XFe96 Extra-cellular Flux Analyzer platform was employed to analyze the mitochondrial oxygen consumption rate (OCR) and extracellular acidification rate (ECAR). The Seahorse software was utilized to analyze the results.

### Transmission electron microscopy section for fibroblasts

The procedure commenced with the removal of the medium from the petri dish, followed by the addition of an electron microscope fixative. After a duration of three hours, the cells were subjected to routine centrifugation. The resulting cell mass was then encapsulated in 1% agarose, and the buffer was rinsed twice for 10 min each. Following this, the fixative was applied for a period of 90 min, after which the buffer was rinsed twice again for 10 min each. Subsequently, the samples underwent dehydration, and a series of acetone and embedding agents were introduced to ensure complete penetration and embedding. Finally, ultra-thin sections approximately 70 nm in thickness were prepared, allowing for observation under a transmission electron microscope using uranium-lead double staining.

### Statistical analysis

All experiments were biologically repeated at least three times. Student’s *t*-test was conducted for two-group comparisons, and one-way analysis of variance (ANOVA) was conducted for three or more group comparisons. GraphPad Prism 7 and SPSS 24.0 were used for data analysis. Data are presented as the average value ± SEM, and *p* values < 0.05 were considered statistically significant.

## Supplementary information

Supplementary Tables

Uncropped western blots

## Data Availability

Bulk RNA-seq data of KIRC and clinical information were downloaded from The Cancer Genome Atlas (TCGA) (the TCGA-KIRC cohort). The datasets generated or analyzed during this study are available from the corresponding author on reasonable request.
