# Supplementary material for: Exosomes derived from ccRCC cells confers fibroblasts activation to foster tumor progression through Warburg effect by downregulating PANK3
Source: Cell Death Discov. 2025 Apr 25;11:198. doi: 10.1038/s41420-025-02434-8 (PMC12032068; doi:10.1038/s41420-025-02434-8)
Supplement: Supplementary file 1 — Supplementary Tables [file 41420_2025_2434_MOESM1_ESM.docx]

**Supplementary Table 1** Summary of Clinicopathologic Variables.

| **Variables** | | **miR-222-3p expression (number of cases)** | | **P-value** |
| --- | --- | --- | --- | --- |
|  |  | **Low (n=112)** | **High (n=94)** |  |
| **Age** |  |  |  |  |
|  | <50 | 70 | 63 | P>0.05 |
|  | ≥50 | 42 | 31 |  |
| **Gender** |  |  |  |  |
|  | Male | 65 | 55 | P>0.05 |
|  | Female | 47 | 39 |  |
| **Tumor size** |  |  |  |  |
|  | <4 | 81 | 73 | P>0.05 |
|  | ≥4 | 31 | 21 |  |
| **Tumor stage** |  |  |  |  |
|  | Localized | 101 | 74 | P<0.05 |
|  | Advanced | 11 | 20 |  |
| **Tumor differentiation** |  |  |  |  |
|  | I-II | 37 | 26 | P>0.05 |
|  | III-IV | 75 | 68 |  |

**Supplementary Table 2** Sequences of primers and siRNA.

| **Gene** | **Sequences** |
| --- | --- |
| IL-1β |  |
| Forward | 5'-ATGATGGCTTATTACAGTGGCAA-3' |
| Reverse | 5'-GTCGGAGATTCGTAGCTGGA-3' |
| IL-6 |  |
| Forward | 5'-ACTCACCTCTTCAGAACGAATTG-3' |
| Reverse | 5'-CCATCTTTGGAAGGTTCAGGTTG-3' |
| IL-8 |  |
| Forward | 5'-TTTTGCCAAGGAGTGCTAAAGA-3' |
| Reverse | 5'-AACCCTCTGCACCCAGTTTTC-3' |
| α-SMA |  |
| Forward | 5'-ACTGCCTTGGTGTGTGACAA-3' |
| Reverse | 5'-TCCCAGTTGGTGATGATGCC-3' |
| FAP |  |
| Forward | 5'-ATGAGCTTCCTCGTCCAATTCA-3' |
| Reverse | 5'-AGACCACCAGAGAGCATATTTTG-3' |
| BBC3 |  |
| Forward | 5'-GCCAGATTTGTGAGACAAGAGG-3' |
| Reverse | 5'-CAGGCACCTAATTGGGCTC-3' |
| DCAF7 |  |
| Forward | 5'-CACGGCAAACGGAAGGAGAT-3' |
| Reverse | 5'-GACGCCTTTTGTGTCAGGGA-3' |
| GNAI3 |  |
| Forward | 5'-ATCGACCGCAACTTACGGG-3' |
| Reverse | 5'-AGTCAATCTTTAGCCGTCCCA-3' |
| HIPK2 |  |
| Forward | 5'-CCCGTGTACGAAGGTATGGC-3' |
| Reverse | 5'-AGTTGGAACTCGGCTCTATTTTC-3' |
| PANK3 |  |
| Forward | 5'-TTTTGGCCGAAGAGGGAACTT-3' |
| Reverse | 5'-TAGCACCGTCTGCAATGTTGA -3' |
| PHACTR4 |  |
| Forward | 5'-GAAGCAGACCAGCCCACTAC-3' |
| Reverse | 5'-CTTGCCAAAGCCTGAGAACTT-3' |
| PPP2R2A |  |
| Forward | 5'-CATACCAGGTGCATGAATACCTC-3' |
| Reverse | 5'-GGGTTATGTCTCGCTTTGTGTTT-3' |
| STOX2 |  |
| Forward | 5'-GACCCGGAGCACAACCTTG-3' |
| Reverse | 5'-GGGAGACATACTGATGGGTGA-3' |
| TLE3 |  |
| Forward | 5'-TATCCGCAGGGCAGACATC-3' |
| Reverse | 5'-GTTTGCCAGCTTGTCGTACTC-3' |
| TRPS1 |  |
| Forward | 5'-AGCCCCAGTAAGGGAGGAAA-3' |
| Reverse | 5'-GGGTGCAGGCCATATCTTGAG-3' |
| UBN2 |  |
| Forward | 5'-GGAGTTCAGTTACCCGGAGC-3' |
| Reverse | 5'-CGGGGTTTCCCACCATATTTC-3' |
| ZFYVE16 |  |
| Forward | 5'-AAGATGTGCCGTCCTCATTGT-3' |
| Reverse | 5'-ATCACCCCGTGCTTTACTTTC-3' |
| ZNF652 |  |
| Forward | 5'-GCTGGTTGAAAACTGTGCTGT-3' |
| Reverse | 5'-GAAGATGGCACTTGACCACGA-3' |
| siPANK3 | 5'-GCAGTCCATTCCAAAGACA-3' |

**Supplementary Table 3** Sequences of miRNA mimics.

| **Mimics** | **Sequences** |
| --- | --- |
| hsa-miR-222-3p |  |
| Forward | 5'-AGCYACAUCUGGCUACUGGGU-3' |
| Reverse | 5'-CCAGUAGCCAGAUGUAGCUUU-3' |
| Mimic negative control |  |
| Forward | 5'-UUCUCCGAACGUGUCACGUTT-3' |
| Reverse | 5'-ACGUGACACGUUCGGAGAATT-3' |
| hsa-miR-222-3p inhibitor | 5'-ACCCAGUAGCCAGAUGUAGCU-3' |
| Inhibitor negative control | 5'-CAGUACUUUUGUGUAGUACAA-3' |
