# Supplementary figures and images for: Exosomes derived from ccRCC cells confers fibroblasts activation to foster tumor progression through Warburg effect by downregulating PANK3
Source: Cell Death Discov. 2025 Apr 25;11:198. doi: 10.1038/s41420-025-02434-8 (PMC12032068; doi:10.1038/s41420-025-02434-8)

Figure 1D

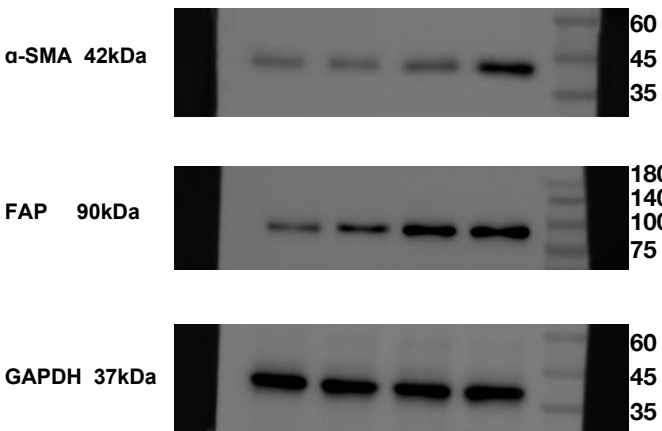

Figure 2B

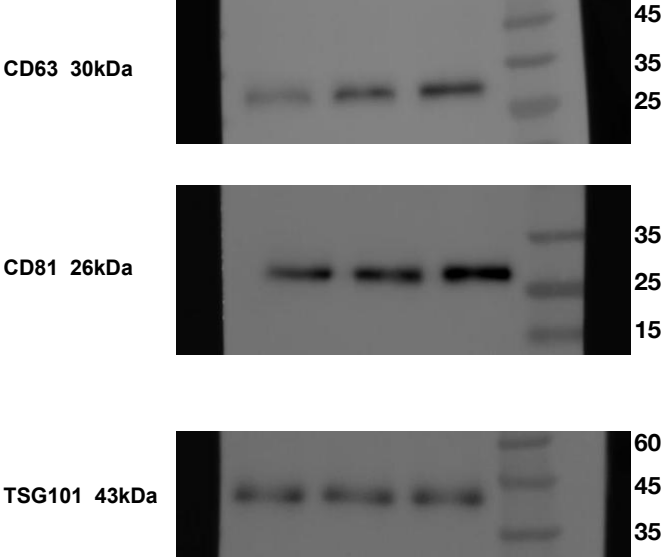

Figure 2D

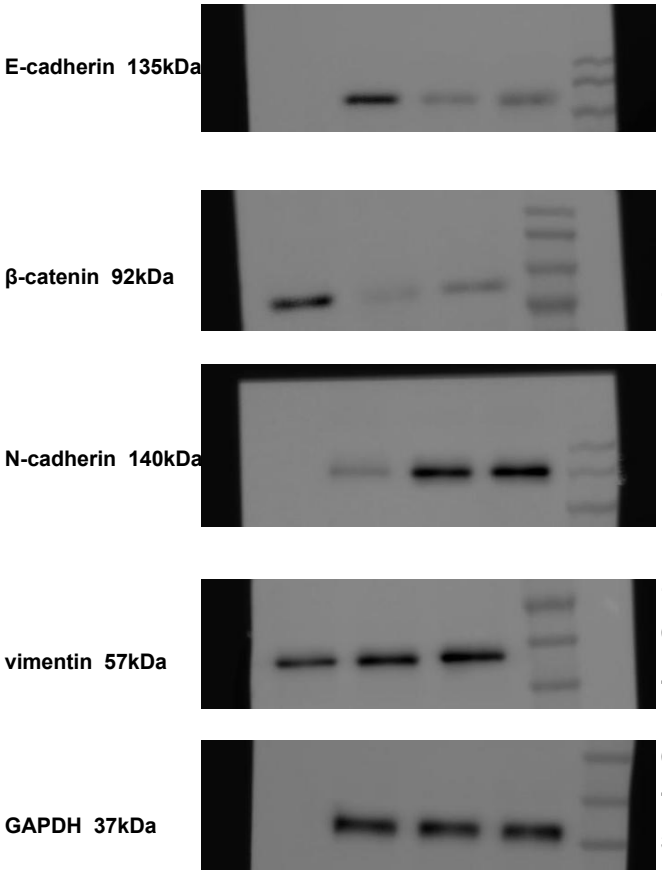

Figure 3H

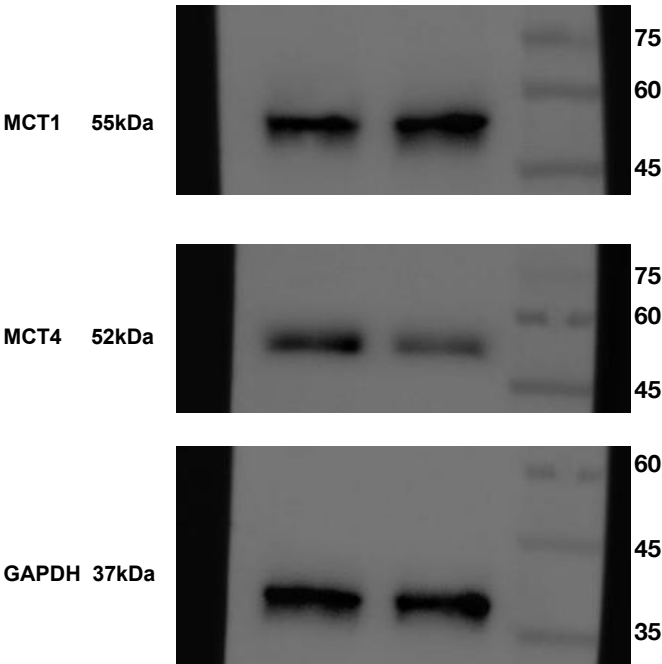

Figure 3I

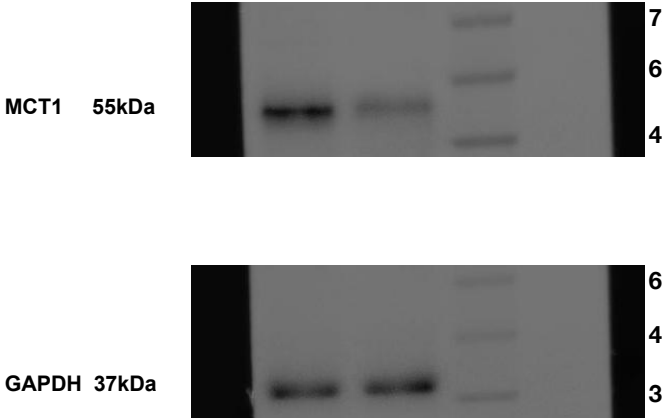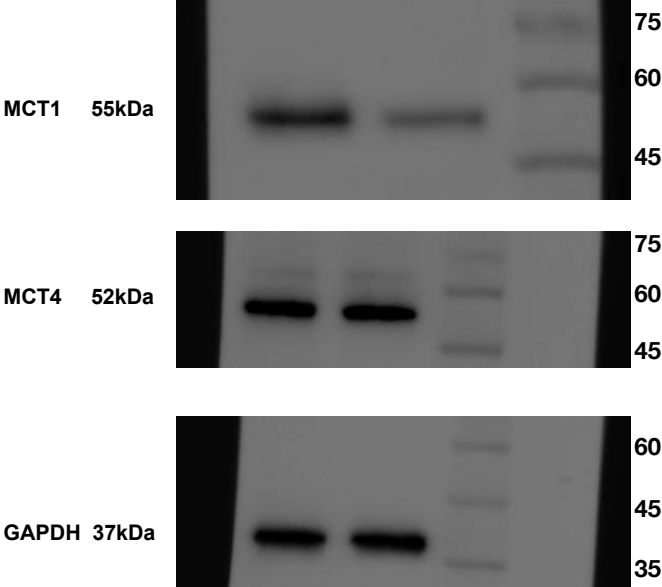

Figure 4D

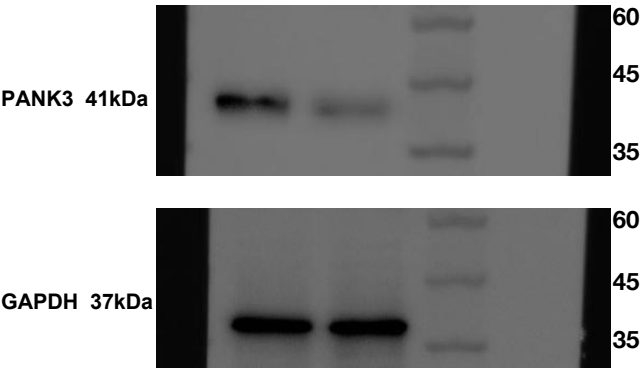

Figure 4E

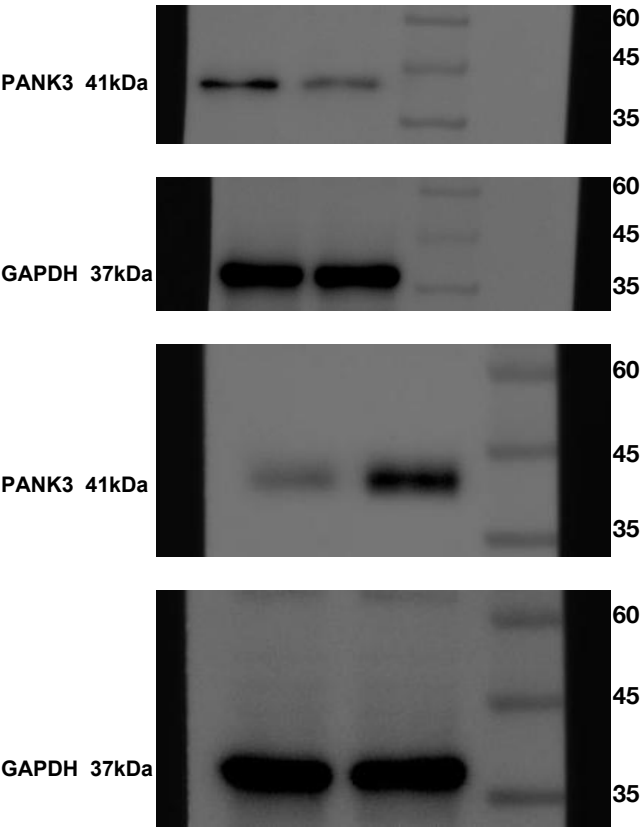

Figure 5K

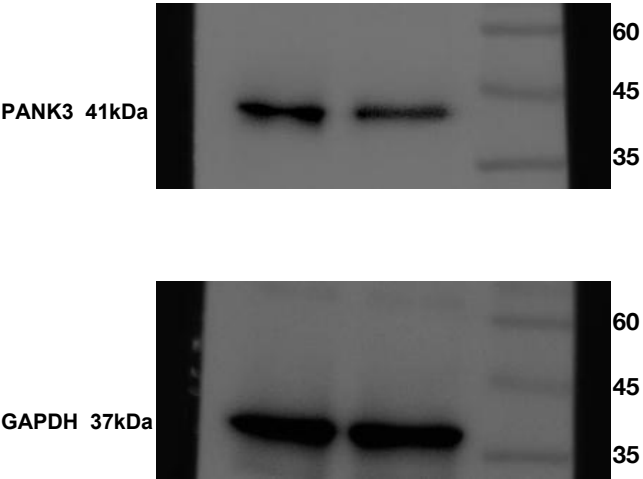

Figure 4I

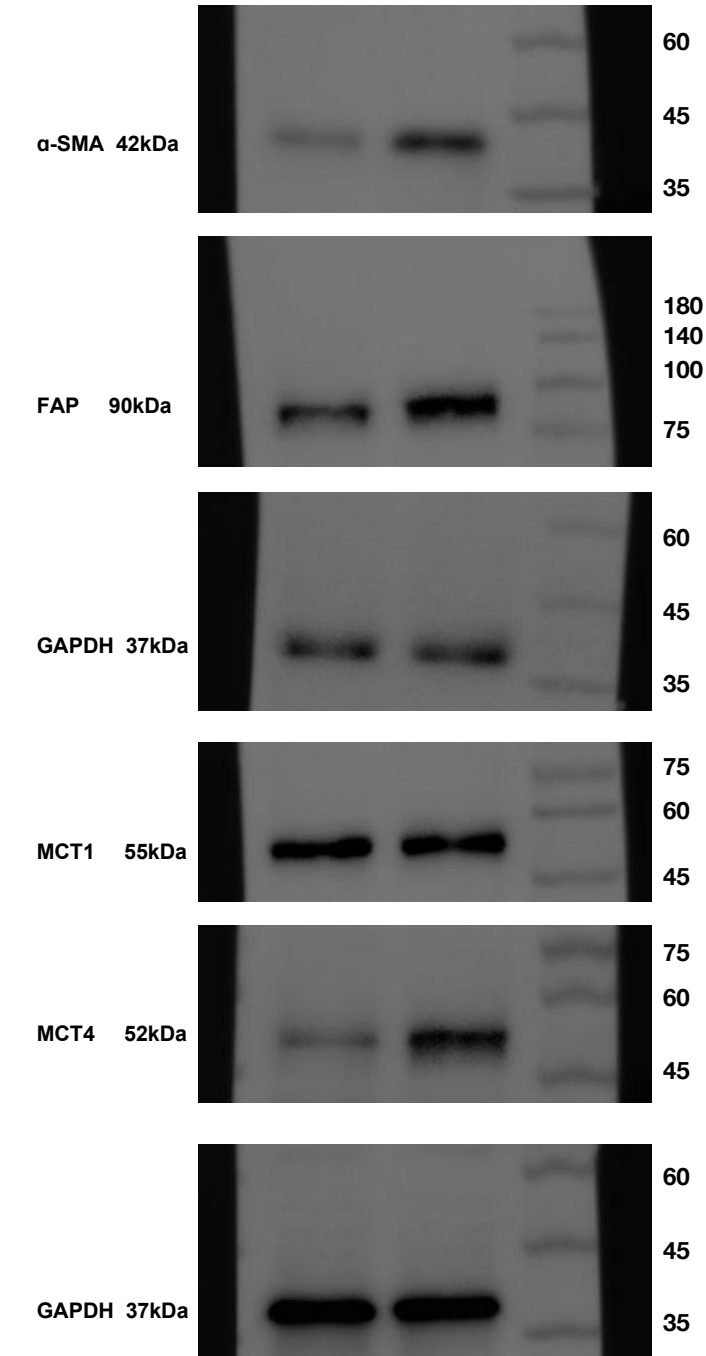

Figure 5O

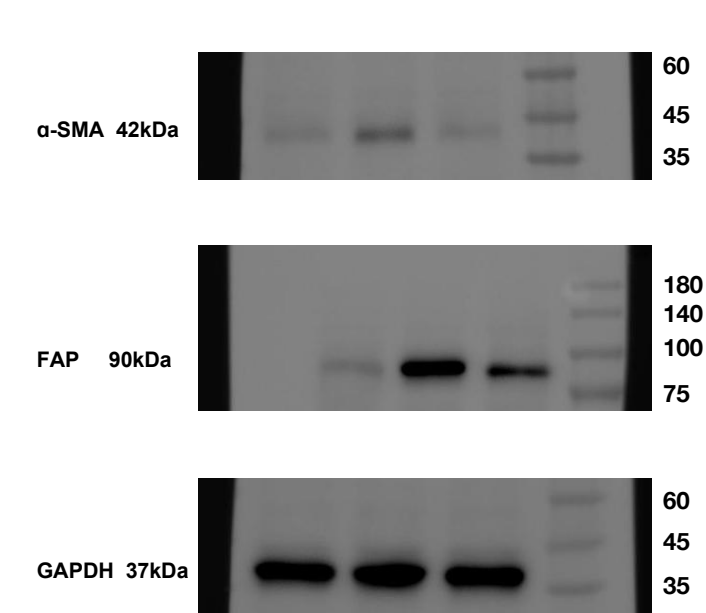

Supplement: Supplementary file 2 — Uncropped western blots [file 41420_2025_2434_MOESM2_ESM.pdf]
